# Supplementary material for: Persistence of Antibiotic-Resistant Escherichia coli Strains Belonging to the B2 Phylogroup in Municipal Wastewater under Aerobic Conditions
Source: Antibiotics (Basel). 2022 Feb 4;11(2):202. doi: 10.3390/antibiotics11020202 (PMC8868233; doi:10.3390/antibiotics11020202)
Supplement: Supplementary file 1 [file antibiotics-11-00202-s001.zip › antibiotics-1565889-supplementary.pdf]

Supplementary Materials

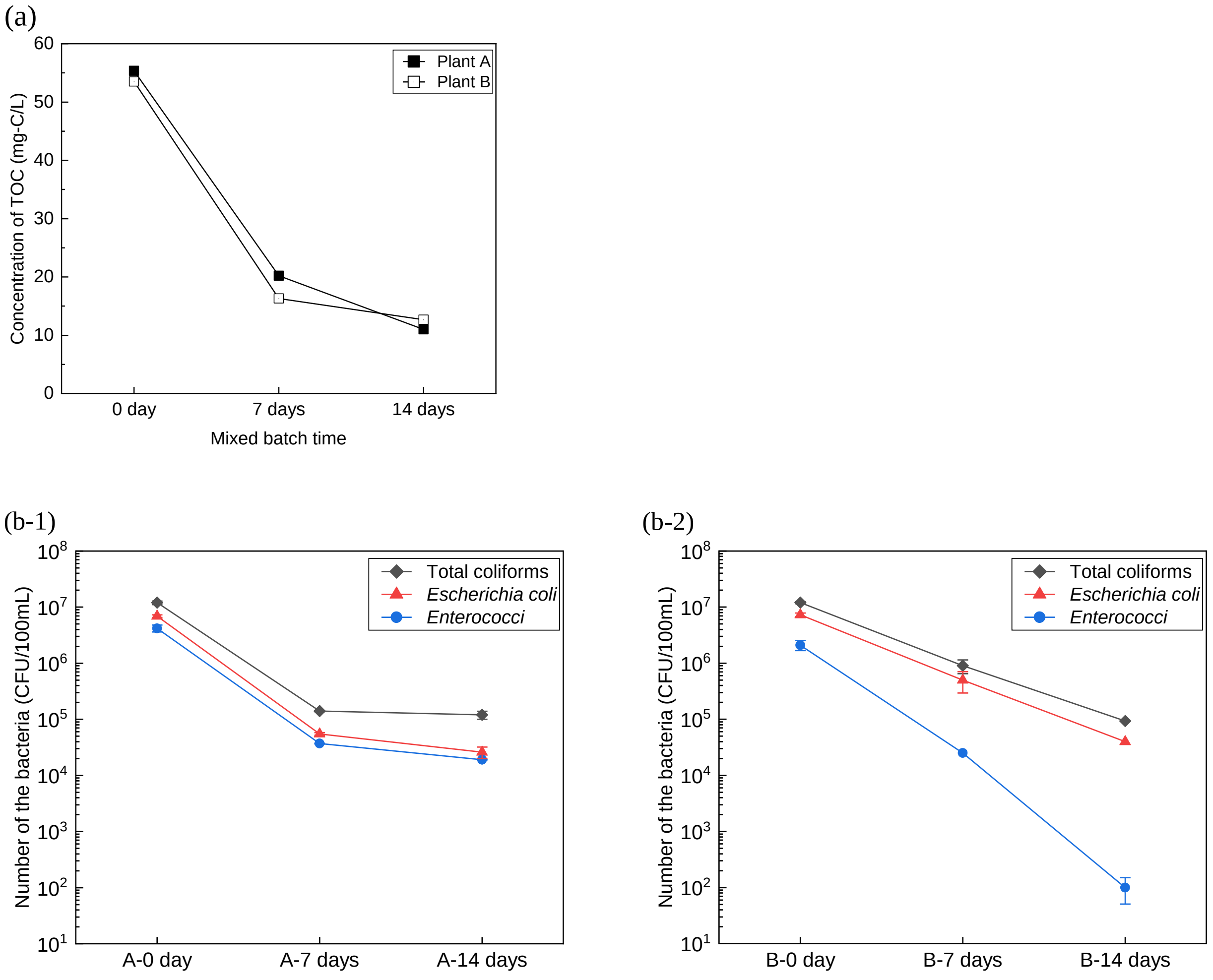

Figure S1. Changes to TOC concentrations and number of bacteria in wastewater samples collected from plants A and B. TOC concentration under aerobic conditions for 14 days (**a**). Abundances of bacteria in wastewater samples collected from plants A (**b-1**) and B (**b-2**).

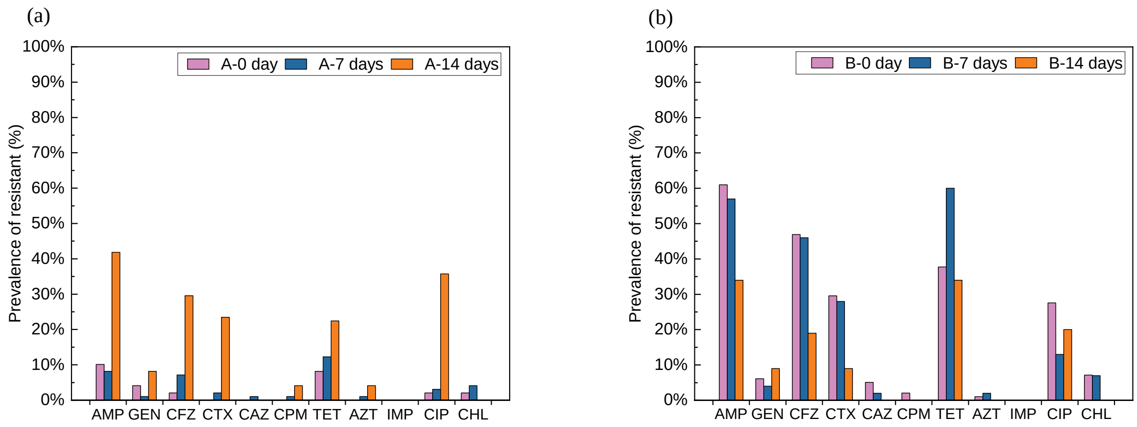

Figure S2: Resistance of *E. coli* isolates in wastewater samples collected from plants A and B to the 11 tested antibiotics. Abbreviations: AMP, ampicillin; GEN, gentamicin; CFZ, cefazolin; CTX, ceftazidime; CAZ, cefotaxime; TET, tetracycline; AZT, Aztreonam; IMP, imipenem; CIP, ciprofloxacin; CPM, Cefepime; CHL, chloramphenicol.

Table S1: Design of primers sequence for detection of phylogroups of *E. coli* isolates.

Primer name	Target	Primer sequences	PCR product (bp)
chuA.1b<br>chuA.2	*chuA*	F: 5’-ATGGTACCGGACGAACCAAC-3’<br>R: 5’-TGCCGCCAGTACCAAAGACA-3’	288
yjaA.1b<br>yjaA.2b	*yjaA*	F: 5’-CAAACGTGAAGTGTCAGGAG-3’<br>R: 5’-AATGCGTTCCTCAACCTGTG-3’	211
TspE4C2.1b<br>TspE4C2.2b	TspE4.C2	F: 5’-CACTATTCGTAAGGTCATCC-3’<br>R: 5’-AGTTTATCGCTGCGGGTCGC-3’	152
AceK.f<br>ArpA1.r	*arpA*	F: 5’-AACGCTATTCGCCAGCTTGC-3’<br>R: 5’-TCTCCCCATACCGTACGCTA-3’	400

Table S2: Design of primers sequence for detection of ESBL-producing genotypes of *E. coli* isolates.

bate-Lactamase(s) genotypes	Primer sequences	PCR product (bp)
TEM-type	F: 5’-CCGTGTCGCCCTTATTCC-3’<br>R: 5’-AGGCACCTATCTCAGCGA-3’	824
SHV-type	F: 5’-ATTTGTCGCTTCTTTACTCGC-3’<br>R: 5’-TTTATGGCGTTACCTTTGACC-3’	1051
CTX-M-1-type	F: 5’-GCTGTTGTTAGGAAGTGTGC-3’<br>R: 5’- CCATTGCCCGAGGTGAAG-3’	516
CTX-M-2-type	F: 5’-ACGCTACCCCTGCTATTT-3’<br>R: 5’- GCTTTCCGCCTTCTGCTC-3’	779 or 780
CTX-M-9-type	F: 5’- GCAGATAATACGCAGGTG-3’<br>R: 5’-CGGCGTGGTGGTGTCTCT-3’	393

Table S3: The parameters of water quality and the number of bacteria in each sample from plant A and B.

Sample	Mixed batch time	pH (-)	EC (µs/cm)	Turbidity (ppm)	TOC (mg·C/L)	Bacteria (CFU/100 mL)		
						Total coliforms	*Escherichia coli*	Enterococci
Plant A	0 day	8.036	0.487	102.40	55.36	1.2×10^{7}	6.9×10^{6}	4.2×10^{6}
	7 days	6.649	0.589	74.14	20.21	1.4×10^{5}	5.5×10^{4}	3.7×10^{4}
	14 days	5.483	0.480	59.10	10.99	1.2×10^{5}	2.6×10^{4}	1.9×10^{4}
Plant B	0 day	7.286	1.640	144.76	53.50	1.2×10^{7}	7.3×10^{6}	2.1×10^{6}
	7 days	6.891	1.723	50.60	16.29	9.0×10^{5}	5.0×10^{5}	2.5×10^{4}
	14 days	5.752	1.675	60.30	12.66	4.0×10^{4}	9.3×10^{4}	1.0×10^{2}

EC, Electrical conductivity; TOC, Total Organic Carbon; N, no data

Table S4. The profiles of antibiotic-resistant *E. coli* isolates in each sample from plant A and B.

Abbreviations: AMP, ampicillin; GEN, gentamicin; CFZ, cefazolin; CTX, ceftazidime; CAZ, cefotaxime; TET, tetracycline; AZT, Aztreonam; IMP, imipenem; CIP, ciprofloxacin; CPM, Cefepime; CHL, chloramphenicol.

Resistance   Intermediate resistance   Susceptibility

Strain code	AMP	GEN	CFZ	CTX	CAZ	TET	IMP	CIP	CHL	CPM	AZT
A-0-1	S≤8	S≤4	S≤2	S≤1	S≤4	S≤4	S≤1	S≤1	S≤8	S≤8	S≤8
A-0-2	S≤8	S≤4	S≤2	S≤1	S≤4	S≤4	S≤1	S≤1	S≤8	S≤8	S≤8
A-0-3	S≤8	S≤4	S≤2	S≤1	S≤4	S≤4	S≤1	S≤1	S≤8	S≤8	S≤8
A-0-4	S≤8	S≤4	S≤2	S≤1	S≤4	S≤4	S≤1	S≤1	S≤8	S≤8	S≤8
A-0-5	S≤8	S≤4	S≤2	S≤1	S≤4	S≤4	S≤1	S≤1	S≤8	S≤8	S≤8
A-0-6	S≤8	S≤4	S≤2	S≤1	S≤4	S≤4	S≤1	S≤1	S≤8	S≤8	S≤8
A-0-7	S≤8	S≤4	S≤2	S≤1	S≤4	S≤4	S≤1	S≤1	S≤8	S≤8	S≤8
A-0-8	S≤8	S≤4	S≤2	S≤1	S≤4	S≤4	S≤1	S≤1	S≤8	S≤8	S≤8
A-0-9	S≤8	S≤4	S≤2	S≤1	S≤4	S≤4	S≤1	S≤1	S≤8	S≤8	S≤8
A-0-10	S≤8	S≤4	S≤2	S≤1	S≤4	S≤4	S≤1	S≤1	S≤8	S≤8	S≤8
A-0-11	S≤8	S≤4	S≤2	S≤1	S≤4	S≤4	S≤1	S≤1	S≤8	S≤8	S≤8
A-0-12	S≤8	S≤4	S≤2	S≤1	S≤4	R≥16	S≤1	S≤1	S≤8	S≤8	S≤8
A-0-13	S≤8	S≤4	S≤2	S≤1	S≤4	R≥16	S≤1	R≥4	R≥32	S≤8	S≤8
A-0-14	S≤8	S≤4	S≤2	S≤1	S≤4	S≤4	S≤1	S≤1	S≤8	S≤8	S≤8
A-0-15	S≤8	S≤4	S≤2	S≤1	S≤4	S≤4	S≤1	S≤1	S≤8	S≤8	S≤8
A-0-16	R≥32	S≤4	R≥8	S≤1	S≤4	R≥16	S≤1	S≤1	S≤8	S≤8	S≤8
A-0-17	S≤8	S≤4	S≤2	S≤1	S≤4	S≤4	S≤1	S≤1	S≤8	S≤8	S≤8
A-0-18	S≤8	S≤4	S≤2	S≤1	S≤4	S≤4	S≤1	S≤1	S≤8	S≤8	S≤8
A-0-19	S≤8	S≤4	S≤2	S≤1	S≤4	S≤4	S≤1	S≤1	S≤8	S≤8	S≤8
A-0-20	S≤8	S≤4	S≤2	S≤1	S≤4	S≤4	S≤1	S≤1	S≤8	S≤8	S≤8
A-0-21	S≤8	S≤4	S≤2	S≤1	S≤4	S≤4	S≤1	S≤1	S≤8	S≤8	S≤8
A-0-22	S≤8	S≤4	S≤2	S≤1	S≤4	S≤4	S≤1	S≤1	S≤8	S≤8	S≤8
A-0-23	S≤8	S≤4	S≤2	S≤1	S≤4	S≤4	S≤1	S≤1	S≤8	S≤8	S≤8
A-0-24	S≤8	S≤4	S≤2	S≤1	S≤4	S≤4	S≤1	S≤1	S≤8	S≤8	S≤8
A-0-25	S≤8	S≤4	S≤2	S≤1	S≤4	S≤4	S≤1	S≤1	S≤8	S≤8	S≤8
A-0-26	S≤8	S≤4	S≤2	S≤1	S≤4	S≤4	S≤1	S≤1	S≤8	S≤8	S≤8
A-0-27	S≤8	S≤4	S≤2	S≤1	S≤4	S≤4	S≤1	S≤1	S≤8	S≤8	S≤8
A-0-28	S≤8	S≤4	S≤2	S≤1	S≤4	S≤4	S≤1	S≤1	S≤8	S≤8	S≤8
A-0-29	S≤8	S≤4	S≤2	S≤1	S≤4	S≤4	S≤1	S≤1	S≤8	S≤8	S≤8
A-0-31	S≤8	S≤4	S≤2	S≤1	S≤4	S≤4	S≤1	S≤1	S≤8	S≤8	S≤8
A-0-32	S≤8	S≤4	S≤2	S≤1	S≤4	S≤4	S≤1	S≤1	S≤8	S≤8	S≤8
A-0-33	S≤8	S≤4	S≤2	S≤1	S≤4	S≤4	S≤1	S≤1	S≤8	S≤8	S≤8
A-0-34	R≥32	R≥16	S≤2	S≤1	S≤4	S≤4	S≤1	S≤1	S≤8	S≤8	S≤8
A-0-35	S≤8	S≤4	S≤2	S≤1	S≤4	S≤4	S≤1	S≤1	S≤8	S≤8	S≤8
A-0-36	S≤8	S≤4	S≤2	S≤1	S≤4	S≤4	S≤1	S≤1	S≤8	S≤8	S≤8
A-0-37	S≤8	S≤4	S≤2	S≤1	S≤4	S≤4	S≤1	S≤1	S≤8	S≤8	S≤8
A-0-38	S≤8	S≤4	S≤2	S≤1	S≤4	S≤4	S≤1	S≤1	S≤8	S≤8	S≤8
A-0-39	S≤8	S≤4	S≤2	S≤1	S≤4	R≥16	S≤1	R≥4	R≥32	S≤8	S≤8
A-0-40	S≤8	S≤4	S≤2	S≤1	S≤4	S≤4	S≤1	S≤1	S≤8	S≤8	S≤8
A-0-41	S≤8	S≤4	S≤2	S≤1	S≤4	S≤4	S≤1	S≤1	S≤8	S≤8	S≤8
A-0-42	S≤8	S≤4	S≤2	S≤1	S≤4	S≤4	S≤1	S≤1	S≤8	S≤8	S≤8
A-0-43	S≤8	S≤4	S≤2	S≤1	S≤4	S≤4	S≤1	S≤1	S≤8	S≤8	S≤8
A-0-44	S≤8	S≤4	S≤2	S≤1	S≤4	S≤4	S≤1	S≤1	S≤8	S≤8	S≤8
A-0-45	S≤8	S≤4	S≤2	S≤1	S≤4	S≤4	S≤1	S≤1	S≤8	S≤8	S≤8
A-0-46	S≤8	S≤4	S≤2	S≤1	S≤4	S≤4	S≤1	S≤1	S≤8	S≤8	S≤8
A-0-47	S≤8	S≤4	S≤2	S≤1	S≤4	S≤4	S≤1	S≤1	S≤8	S≤8	S≤8
A-0-48	S≤8	S≤4	S≤2	S≤1	S≤4	S≤4	S≤1	S≤1	S≤8	S≤8	S≤8
A-0-49	S≤8	S≤4	S≤2	S≤1	S≤4	S≤4	S≤1	S≤1	S≤8	S≤8	S≤8
A-0-50	S≤8	S≤4	S≤2	S≤1	S≤4	S≤4	S≤1	S≤1	S≤8	S≤8	S≤8
A-0-51	R≥32	S≤4	S≤2	S≤1	S≤4	R≥16	S≤1	I=2	S≤8	S≤8	S≤8
A-0-52	S≤8	S≤4	S≤2	S≤1	S≤4	S≤4	S≤1	S≤1	S≤8	S≤8	S≤8
A-0-53	S≤8	S≤4	S≤2	S≤1	S≤4	S≤4	S≤1	S≤1	S≤8	S≤8	S≤8
A-0-54	S≤8	S≤4	S≤2	S≤1	S≤4	S≤4	S≤1	S≤1	S≤8	S≤8	S≤8
A-0-55	S≤8	S≤4	S≤2	S≤1	S≤4	S≤4	S≤1	S≤1	S≤8	S≤8	S≤8
A-0-56	S≤8	S≤4	S≤2	S≤1	S≤4	S≤4	S≤1	S≤1	S≤8	S≤8	S≤8
A-0-57	S≤8	S≤4	S≤2	S≤1	S≤4	S≤4	S≤1	S≤1	S≤8	S≤8	S≤8
A-0-58	S≤8	S≤4	S≤2	S≤1	S≤4	S≤4	S≤1	S≤1	S≤8	S≤8	S≤8
A-0-59	S≤8	S≤4	S≤2	S≤1	S≤4	S≤4	S≤1	S≤1	S≤8	S≤8	S≤8
A-0-60	R≥32	S≤4	S≤2	S≤1	S≤4	S≤4	S≤1	S≤1	S≤8	S≤8	S≤8
A-0-61	S≤8	S≤4	S≤2	S≤1	S≤4	S≤4	S≤1	S≤1	S≤8	S≤8	S≤8
A-0-62	S≤8	S≤4	S≤2	S≤1	S≤4	S≤4	S≤1	S≤1	S≤8	S≤8	S≤8
A-0-63	S≤8	S≤4	S≤2	S≤1	S≤4	S≤4	S≤1	S≤1	S≤8	S≤8	S≤8
A-0-64	S≤8	S≤4	S≤2	S≤1	S≤4	S≤4	S≤1	S≤1	S≤8	S≤8	S≤8
A-0-65	S≤8	S≤4	S≤2	S≤1	S≤4	S≤4	S≤1	S≤1	S≤8	S≤8	S≤8
A-0-66	S≤8	S≤4	S≤2	S≤1	S≤4	S≤4	S≤1	S≤1	S≤8	S≤8	S≤8
A-0-67	S≤8	S≤4	S≤2	S≤1	S≤4	S≤4	S≤1	S≤1	S≤8	S≤8	S≤8
A-0-68	R≥32	R≥16	I=4	S≤1	S≤4	R≥16	S≤1	S≤1	S≤8	S≤8	S≤8
A-0-69	R≥32	R≥16	I=4	S≤1	S≤4	R≥16	S≤1	S≤1	S≤8	S≤8	S≤8
A-0-70	S≤8	S≤4	S≤2	S≤1	S≤4	S≤4	S≤1	S≤1	S≤8	S≤8	S≤8
A-0-71	S≤8	S≤4	S≤2	S≤1	S≤4	S≤4	S≤1	S≤1	S≤8	S≤8	S≤8
A-0-72	S≤8	S≤4	S≤2	S≤1	S≤4	S≤4	S≤1	S≤1	S≤8	S≤8	S≤8
A-0-73	S≤8	S≤4	S≤2	S≤1	S≤4	S≤4	S≤1	S≤1	S≤8	S≤8	S≤8
A-0-74	S≤8	S≤4	S≤2	S≤1	S≤4	S≤4	S≤1	S≤1	S≤8	S≤8	S≤8
A-0-75	S≤8	S≤4	S≤2	S≤1	S≤4	S≤4	S≤1	S≤1	S≤8	S≤8	S≤8
A-0-76	S≤8	S≤4	S≤2	S≤1	S≤4	S≤4	S≤1	S≤1	S≤8	S≤8	S≤8
A-0-77	S≤8	S≤4	S≤2	S≤1	S≤4	S≤4	S≤1	S≤1	S≤8	S≤8	S≤8
A-0-78	S≤8	S≤4	S≤2	S≤1	S≤4	S≤4	S≤1	S≤1	S≤8	S≤8	S≤8
A-0-79	S≤8	S≤4	S≤2	S≤1	S≤4	S≤4	S≤1	S≤1	S≤8	S≤8	S≤8
A-0-80	R≥32	S≤4	S≤2	S≤1	S≤4	S≤4	S≤1	S≤1	S≤8	S≤8	S≤8
A-0-81	S≤8	S≤4	S≤2	S≤1	S≤4	R≥16	S≤1	S≤1	S≤8	S≤8	S≤8
A-0-82	S≤8	S≤4	S≤2	S≤1	S≤4	S≤4	S≤1	S≤1	S≤8	S≤8	S≤8
A-0-83	S≤8	S≤4	S≤2	S≤1	S≤4	S≤4	S≤1	S≤1	S≤8	S≤8	S≤8
A-0-84	S≤8	S≤4	S≤2	S≤1	S≤4	S≤4	S≤1	S≤1	S≤8	S≤8	S≤8
A-0-85	S≤8	S≤4	S≤2	S≤1	S≤4	S≤4	S≤1	S≤1	S≤8	S≤8	S≤8
A-0-86	S≤8	S≤4	S≤2	S≤1	S≤4	S≤4	S≤1	S≤1	S≤8	S≤8	S≤8
A-0-87	S≤8	S≤4	S≤2	S≤1	S≤4	S≤4	S≤1	S≤1	S≤8	S≤8	S≤8
A-0-88	S≤8	S≤4	S≤2	S≤1	S≤4	S≤4	S≤1	S≤1	S≤8	S≤8	S≤8
A-0-89	S≤8	S≤4	S≤2	S≤1	S≤4	S≤4	S≤1	S≤1	S≤8	S≤8	S≤8
A-0-90	S≤8	S≤4	S≤2	S≤1	S≤4	S≤4	S≤1	S≤1	S≤8	S≤8	S≤8
A-0-91	S≤8	S≤4	S≤2	S≤1	S≤4	S≤4	S≤1	S≤1	S≤8	S≤8	S≤8
A-0-92	R≥32	R≥16	S≤2	S≤1	S≤4	S≤4	S≤1	S≤1	S≤8	S≤8	S≤8
A-0-93	S≤8	S≤4	S≤2	S≤1	S≤4	S≤4	S≤1	S≤1	S≤8	S≤8	S≤8
A-0-94	S≤8	S≤4	S≤2	S≤1	S≤4	S≤4	S≤1	S≤1	S≤8	S≤8	S≤8
A-0-95	S≤8	S≤4	S≤2	S≤1	S≤4	S≤4	S≤1	S≤1	S≤8	S≤8	S≤8
A-0-96	S≤8	S≤4	S≤2	S≤1	S≤4	S≤4	S≤1	S≤1	S≤8	S≤8	S≤8
A-0-97	R≥32	S≤4	S≤2	S≤1	S≤4	S≤4	S≤1	S≤1	S≤8	S≤8	S≤8
A-0-98	R≥32	S≤4	S≤2	S≤1	S≤4	S≤4	S≤1	S≤1	S≤8	S≤8	S≤8
A-0-99	S≤8	S≤4	S≤2	S≤1	S≤4	S≤4	S≤1	S≤1	S≤8	S≤8	S≤8
A-0-100	S≤8	S≤4	S≤2	S≤1	S≤4	S≤4	S≤1	S≤1	S≤8	S≤8	S≤8

Strain code	AMP	GEN	CFZ	CTX	CAZ	TET	IMP	CIP	CHL	CPM	AZT
A-7-1	S≤8	S≤4	S≤2	S≤1	S≤4	S≤4	S≤1	S≤1	S≤8	S≤8	S≤8
A-7-2	S≤8	S≤4	S≤2	S≤1	S≤4	S≤4	S≤1	S≤1	S≤8	S≤8	S≤8
A-7-3	S≤8	S≤4	S≤2	S≤1	S≤4	S≤4	S≤1	S≤1	S≤8	S≤8	S≤8
A-7-4	S≤8	S≤4	S≤2	S≤1	S≤4	S≤4	S≤1	S≤1	S≤8	S≤8	S≤8
A-7-5	S≤8	S≤4	S≤2	S≤1	S≤4	S≤4	S≤1	S≤1	S≤8	S≤8	S≤8
A-7-6	S≤8	S≤4	S≤2	S≤1	S≤4	S≤4	S≤1	S≤1	S≤8	S≤8	S≤8
A-7-7	S≤8	S≤4	S≤2	S≤1	S≤4	S≤4	S≤1	S≤1	S≤8	S≤8	S≤8
A-7-8	S≤8	S≤4	S≤2	S≤1	S≤4	S≤4	S≤1	S≤1	S≤8	S≤8	S≤8
A-7-9	S≤8	S≤4	S≤2	S≤1	S≤4	S≤4	S≤1	S≤1	S≤8	S≤8	S≤8
A-7-10	S≤8	S≤4	S≤2	S≤1	S≤4	S≤4	S≤1	S≤1	S≤8	S≤8	S≤8
A-7-11	S≤8	S≤4	S≤2	S≤1	S≤4	S≤4	S≤1	S≤1	S≤8	S≤8	S≤8
A-7-13	S≤8	S≤4	S≤2	S≤1	S≤4	S≤4	S≤1	S≤1	S≤8	S≤8	S≤8
A-7-14	S≤8	S≤4	S≤2	S≤1	S≤4	S≤4	S≤1	S≤1	S≤8	S≤8	S≤8
A-7-15	S≤8	S≤4	S≤2	S≤1	S≤4	S≤4	S≤1	S≤1	S≤8	S≤8	S≤8
A-7-16	S≤8	S≤4	S≤2	S≤1	S≤4	S≤4	S≤1	S≤1	S≤8	S≤8	S≤8
A-7-17	S≤8	S≤4	S≤2	S≤1	S≤4	S≤4	S≤1	S≤1	S≤8	S≤8	S≤8
A-7-18	S≤8	S≤4	S≤2	S≤1	S≤4	S≤4	S≤1	S≤1	S≤8	S≤8	S≤8
A-7-19	S≤8	S≤4	S≤2	S≤1	S≤4	R≥16	S≤1	R≥4	S≤8	S≤8	S≤8
A-7-20	S≤8	S≤4	S≤2	S≤1	S≤4	S≤4	S≤1	S≤1	S≤8	S≤8	S≤8
A-7-21	S≤8	S≤4	I=4	S≤1	S≤4	S≤4	S≤1	S≤1	S≤8	S≤8	S≤8
A-7-22	S≤8	S≤4	S≤2	S≤1	S≤4	S≤4	S≤1	S≤1	S≤8	S≤8	S≤8
A-7-23	S≤8	S≤4	S≤2	S≤1	S≤4	S≤4	S≤1	S≤1	S≤8	S≤8	S≤8
A-7-24	S≤8	S≤4	S≤2	S≤1	S≤4	S≤4	S≤1	S≤1	S≤8	S≤8	S≤8
A-7-25	S≤8	S≤4	S≤2	S≤1	S≤4	S≤4	S≤1	S≤1	S≤8	S≤8	S≤8
A-7-26	S≤8	S≤4	S≤2	S≤1	S≤4	S≤4	S≤1	S≤1	S≤8	S≤8	S≤8
A-7-27	S≤8	S≤4	S≤2	S≤1	S≤4	S≤4	S≤1	S≤1	S≤8	S≤8	S≤8
A-7-28	S≤8	S≤4	S≤2	S≤1	S≤4	S≤4	S≤1	S≤1	S≤8	S≤8	S≤8
A-7-29	S≤8	S≤4	S≤2	S≤1	S≤4	S≤4	S≤1	S≤1	S≤8	S≤8	S≤8
A-7-30	S≤8	S≤4	S≤2	S≤1	S≤4	S≤4	S≤1	S≤1	S≤8	S≤8	S≤8
A-7-31	S≤8	S≤4	S≤2	S≤1	S≤4	S≤4	S≤1	S≤1	S≤8	S≤8	S≤8
A-7-32	S≤8	S≤4	S≤2	S≤1	S≤4	S≤4	S≤1	S≤1	S≤8	S≤8	S≤8
A-7-34	S≤8	S≤4	S≤2	S≤1	S≤4	S≤4	S≤1	S≤1	S≤8	S≤8	S≤8
A-7-35	R≥32	S≤4	R≥8	I=2	I=8	R≥16	S≤1	S≤1	S≤8	S≤8	S≤8
A-7-36	S≤8	S≤4	S≤2	S≤1	S≤4	S≤4	S≤1	S≤1	S≤8	S≤8	S≤8
A-7-37	S≤8	S≤4	S≤2	S≤1	S≤4	S≤4	S≤1	S≤1	S≤8	S≤8	S≤8
A-7-38	S≤8	S≤4	S≤2	S≤1	S≤4	S≤4	S≤1	S≤1	S≤8	S≤8	S≤8
A-7-39	S≤8	S≤4	S≤2	S≤1	S≤4	R≥16	S≤1	S≤1	S≤8	S≤8	S≤8
A-7-40	R≥32	S≤4	S≤2	S≤1	S≤4	S≤4	S≤1	S≤1	S≤8	S≤8	S≤8
A-7-41	S≤8	S≤4	S≤2	S≤1	S≤4	S≤4	S≤1	S≤1	S≤8	S≤8	S≤8
A-7-42	S≤8	S≤4	S≤2	S≤1	S≤4	S≤4	S≤1	S≤1	S≤8	S≤8	S≤8
A-7-43	S≤8	S≤4	S≤2	S≤1	S≤4	S≤4	S≤1	S≤1	S≤8	S≤8	S≤8
A-7-44	S≤8	S≤4	S≤2	S≤1	S≤4	S≤4	S≤1	S≤1	S≤8	S≤8	S≤8
A-7-45	S≤8	S≤4	S≤2	S≤1	S≤4	S≤4	S≤1	S≤1	S≤8	S≤8	S≤8
A-7-46	S≤8	S≤4	S≤2	S≤1	S≤4	S≤4	S≤1	S≤1	S≤8	S≤8	S≤8
A-7-47	S≤8	S≤4	S≤2	S≤1	S≤4	S≤4	S≤1	S≤1	S≤8	S≤8	S≤8
A-7-48	R≥32	R≥16	R≥8	S≤1	S≤4	R≥16	S≤1	S≤1	S≤8	S≤8	S≤8
A-7-49	I=16	S≤4	S≤2	S≤1	S≤4	S≤4	S≤1	S≤1	S≤8	S≤8	S≤8
A-7-50	S≤8	S≤4	S≤2	S≤1	S≤4	R≥16	S≤1	S≤1	R≥32	S≤8	S≤8
A-7-51	I=16	S≤4	S≤2	S≤1	S≤4	S≤4	S≤1	S≤1	S≤8	S≤8	S≤8
A-7-52	R≥32	S≤4	S≤2	S≤1	S≤4	R≥16	S≤1	S≤1	S≤8	S≤8	S≤8
A-7-53	S≤8	S≤4	S≤2	S≤1	S≤4	S≤4	S≤1	S≤1	S≤8	S≤8	S≤8
A-7-54	S≤8	S≤4	S≤2	S≤1	S≤4	S≤4	S≤1	S≤1	S≤8	S≤8	S≤8
A-7-55	S≤8	S≤4	S≤2	S≤1	S≤4	S≤4	S≤1	S≤1	S≤8	S≤8	S≤8
A-7-56	S≤8	S≤4	S≤2	S≤1	S≤4	R≥16	S≤1	S≤1	S≤8	S≤8	S≤8
A-7-57	S≤8	S≤4	S≤2	S≤1	S≤4	S≤4	S≤1	S≤1	S≤8	S≤8	S≤8
A-7-58	S≤8	S≤4	S≤2	S≤1	S≤4	S≤4	S≤1	S≤1	S≤8	S≤8	S≤8
A-7-59	S≤8	S≤4	S≤2	S≤1	S≤4	S≤4	S≤1	S≤1	S≤8	S≤8	S≤8
A-7-60	S≤8	S≤4	S≤2	S≤1	S≤4	S≤4	S≤1	S≤1	S≤8	S≤8	S≤8
A-7-61	S≤8	S≤4	S≤2	S≤1	S≤4	S≤4	S≤1	S≤1	S≤8	S≤8	S≤8
A-7-62	S≤8	S≤4	S≤2	S≤1	S≤4	S≤4	S≤1	S≤1	S≤8	S≤8	S≤8
A-7-63	S≤8	S≤4	S≤2	S≤1	S≤4	S≤4	S≤1	S≤1	S≤8	S≤8	S≤8
A-7-64	S≤8	S≤4	S≤2	S≤1	S≤4	S≤4	S≤1	S≤1	S≤8	S≤8	S≤8
A-7-65	S≤8	S≤4	S≤2	S≤1	S≤4	S≤4	S≤1	S≤1	S≤8	S≤8	S≤8
A-7-66	S≤8	S≤4	S≤2	S≤1	S≤4	S≤4	S≤1	S≤1	S≤8	S≤8	S≤8
A-7-67	S≤8	S≤4	S≤2	S≤1	S≤4	S≤4	S≤1	S≤1	S≤8	S≤8	S≤8
A-7-68	S≤8	S≤4	S≤2	S≤1	S≤4	S≤4	S≤1	S≤1	S≤8	S≤8	S≤8
A-7-69	S≤8	S≤4	S≤2	S≤1	S≤4	S≤4	S≤1	S≤1	S≤8	S≤8	S≤8
A-7-70	S≤8	S≤4	S≤2	S≤1	S≤4	S≤4	S≤1	S≤1	S≤8	S≤8	S≤8
A-7-71	S≤8	S≤4	S≤2	S≤1	S≤4	S≤4	S≤1	S≤1	S≤8	S≤8	S≤8
A-7-72	S≤8	S≤4	S≤2	S≤1	S≤4	S≤4	S≤1	S≤1	S≤8	S≤8	S≤8
A-7-73	S≤8	S≤4	S≤2	S≤1	S≤4	S≤4	S≤1	S≤1	S≤8	S≤8	S≤8
A-7-74	S≤8	S≤4	S≤2	S≤1	S≤4	S≤4	S≤1	S≤1	S≤8	S≤8	S≤8
A-7-75	S≤8	S≤4	S≤2	S≤1	S≤4	S≤4	S≤1	S≤1	S≤8	S≤8	S≤8
A-7-76	R≥32	S≤4	R≥8	S≤1	S≤4	R≥16	S≤1	S≤1	R≥32	S≤8	S≤8
A-7-77	S≤8	S≤4	S≤2	S≤1	S≤4	S≤4	S≤1	S≤1	S≤8	S≤8	S≤8
A-7-78	S≤8	S≤4	S≤2	S≤1	S≤4	S≤4	S≤1	S≤1	S≤8	S≤8	S≤8
A-7-79	S≤8	S≤4	S≤2	S≤1	S≤4	S≤4	S≤1	S≤1	S≤8	S≤8	S≤8
A-7-80	S≤8	S≤4	S≤2	S≤1	S≤4	S≤4	S≤1	S≤1	S≤8	S≤8	S≤8
A-7-81	S≤8	S≤4	S≤2	S≤1	S≤4	S≤4	S≤1	S≤1	S≤8	S≤8	S≤8
A-7-82	S≤8	S≤4	S≤2	S≤1	S≤4	S≤4	S≤1	S≤1	S≤8	S≤8	S≤8
A-7-83	S≤8	S≤4	S≤2	S≤1	S≤4	S≤4	S≤1	S≤1	S≤8	S≤8	S≤8
A-7-84	S≤8	S≤4	S≤2	S≤1	S≤4	S≤4	S≤1	S≤1	S≤8	S≤8	S≤8
A-7-85	S≤8	S≤4	S≤2	S≤1	S≤4	S≤4	S≤1	S≤1	S≤8	S≤8	S≤8
A-7-86	S≤8	S≤4	S≤2	S≤1	S≤4	R≥16	S≤1	S≤1	S≤8	S≤8	S≤8
A-7-87	S≤8	S≤4	S≤2	S≤1	S≤4	S≤4	S≤1	S≤1	S≤8	S≤8	S≤8
A-7-88	S≤8	S≤4	S≤2	S≤1	S≤4	S≤4	S≤1	S≤1	S≤8	S≤8	S≤8
A-7-89	S≤8	S≤4	S≤2	S≤1	S≤4	S≤4	S≤1	S≤1	S≤8	S≤8	S≤8
A-7-90	S≤8	S≤4	S≤2	S≤1	S≤4	S≤4	S≤1	S≤1	S≤8	S≤8	S≤8
A-7-91	R≥32	S≤4	R≥8	R≥4	S≤4	R≥16	S≤1	R≥4	S≤8	I=16	I=16
A-7-92	R≥32	S≤4	S≤2	S≤1	S≤4	S≤4	S≤1	S≤1	S≤8	S≤8	S≤8
A-7-93	S≤8	S≤4	S≤2	S≤1	S≤4	S≤4	S≤1	S≤1	S≤8	S≤8	S≤8
A-7-94	S≤8	S≤4	S≤2	S≤1	S≤4	S≤4	S≤1	R≥4	S≤8	S≤8	S≤8
A-7-95	S≤8	S≤4	S≤2	S≤1	S≤4	S≤4	S≤1	S≤1	S≤8	S≤8	S≤8
A-7-96	S≤8	S≤4	S≤2	S≤1	S≤4	S≤4	S≤1	S≤1	S≤8	S≤8	S≤8
A-7-97	S≤8	S≤4	S≤2	S≤1	S≤4	R≥16	S≤1	S≤1	R≥32	S≤8	S≤8
A-7-98	S≤8	S≤4	I=4	S≤1	S≤4	S≤4	S≤1	S≤1	S≤8	S≤8	S≤8
A-7-99	S≤8	S≤4	S≤2	S≤1	S≤4	R≥16	S≤1	S≤1	R≥32	S≤8	S≤8
A-7-100	R≥32	S≤4	R≥8	R≥4	R≥16	S≤4	S≤1	S≤1	S≤8	R≥32	R≥32

Strain code	AMP	GEN	CFZ	CTX	CAZ	TET	IMP	CIP	CHL	CPM	AZT
A-14-1	S≤8	S≤4	S≤2	S≤1	S≤4	S≤4	S≤1	S≤1	S≤8	S≤8	S≤8
A-14-2	S≤8	S≤4	S≤2	S≤1	S≤4	S≤4	S≤1	S≤1	S≤8	S≤8	S≤8
A-14-3	S≤8	S≤4	S≤2	S≤1	S≤4	S≤4	S≤1	S≤1	S≤8	S≤8	S≤8
A-14-4	S≤8	S≤4	S≤2	S≤1	S≤4	S≤4	S≤1	S≤1	S≤8	S≤8	S≤8
A-14-5	R≥32	R≥16	I=4	S≤1	S≤4	S≤4	S≤1	R≥4	S≤8	S≤8	S≤8
A-14-6	S≤8	S≤4	S≤2	S≤1	S≤4	S≤4	S≤1	S≤1	S≤8	S≤8	S≤8
A-14-7	R≥32	S≤4	R≥8	R≥4	S≤4	R≥16	S≤1	R≥4	S≤8	S≤8	S≤8
A-14-8	R≥32	S≤4	R≥8	R≥4	S≤4	R≥16	S≤1	R≥4	S≤8	S≤8	I=16
A-14-9	R≥32	S≤4	R≥8	R≥4	S≤4	R≥16	S≤1	R≥4	S≤8	S≤8	S≤8
A-14-10	S≤8	S≤4	S≤2	S≤1	S≤4	S≤4	S≤1	R≥4	S≤8	S≤8	S≤8
A-14-11	S≤8	S≤4	S≤2	S≤1	S≤4	S≤4	S≤1	S≤1	S≤8	S≤8	S≤8
A-14-12	S≤8	S≤4	S≤2	S≤1	S≤4	S≤4	S≤1	S≤1	S≤8	S≤8	S≤8
A-14-13	S≤8	S≤4	S≤2	S≤1	S≤4	S≤4	S≤1	R≥4	S≤8	S≤8	S≤8
A-14-14	R≥32	R≥16	S≤2	S≤1	S≤4	S≤4	S≤1	R≥4	S≤8	S≤8	S≤8
A-14-15	S≤8	S≤4	S≤2	S≤1	S≤4	S≤4	S≤1	S≤1	S≤8	S≤8	S≤8
A-14-16	S≤8	S≤4	S≤2	S≤1	S≤4	S≤4	S≤1	S≤1	S≤8	S≤8	S≤8
A-14-17	R≥32	R≥16	S≤2	S≤1	S≤4	S≤4	S≤1	R≥4	S≤8	S≤8	S≤8
A-14-18	R≥32	R≥16	S≤2	S≤1	S≤4	S≤4	S≤1	R≥4	S≤8	S≤8	S≤8
A-14-19	R≥32	R≥16	S≤2	S≤1	S≤4	S≤4	S≤1	R≥4	S≤8	S≤8	S≤8
A-14-20	R≥32	S≤4	I=4	S≤1	S≤4	R≥16	S≤1	R≥4	S≤8	S≤8	S≤8
A-14-21	R≥32	S≤4	R≥8	R≥4	S≤4	R≥16	S≤1	R≥4	S≤8	R≥32	R≥32
A-14-22	S≤8	S≤4	S≤2	S≤1	S≤4	S≤4	S≤1	S≤1	S≤8	S≤8	S≤8
A-14-23	R≥32	S≤4	R≥8	R≥4	S≤4	R≥16	S≤1	R≥4	S≤8	S≤8	S≤8
A-14-24	S≤8	S≤4	S≤2	S≤1	S≤4	S≤4	S≤1	S≤1	S≤8	S≤8	S≤8
A-14-25	S≤8	S≤4	S≤2	S≤1	S≤4	S≤4	S≤1	S≤1	S≤8	S≤8	S≤8
A-14-26	S≤8	S≤4	S≤2	S≤1	S≤4	S≤4	S≤1	S≤1	S≤8	S≤8	S≤8
A-14-27	S≤8	S≤4	S≤2	S≤1	S≤4	S≤4	S≤1	S≤1	S≤8	S≤8	S≤8
A-14-28	S≤8	S≤4	S≤2	S≤1	S≤4	S≤4	S≤1	S≤1	S≤8	S≤8	S≤8
A-14-29	S≤8	S≤4	S≤2	S≤1	S≤4	S≤4	S≤1	S≤1	S≤8	S≤8	S≤8
A-14-30	R≥32	S≤4	S≤2	S≤1	S≤4	R≥16	S≤1	R≥4	S≤8	S≤8	S≤8
A-14-31	S≤8	S≤4	S≤2	S≤1	S≤4	S≤4	S≤1	S≤1	S≤8	S≤8	S≤8
A-14-32	S≤8	S≤4	S≤2	S≤1	S≤4	S≤4	S≤1	S≤1	S≤8	S≤8	S≤8
A-14-33	S≤8	S≤4	S≤2	S≤1	S≤4	R≥16	S≤1	S≤1	S≤8	S≤8	S≤8
A-14-34	S≤8	S≤4	S≤2	S≤1	S≤4	S≤4	S≤1	S≤1	S≤8	S≤8	S≤8
A-14-35	R≥32	S≤4	R≥8	S≤1	I=8	S≤4	S≤1	S≤1	S≤8	S≤8	S≤8
A-14-36	S≤8	S≤4	S≤2	S≤1	S≤4	S≤4	S≤1	S≤1	S≤8	S≤8	S≤8
A-14-37	S≤8	S≤4	S≤2	S≤1	S≤4	S≤4	S≤1	S≤1	S≤8	S≤8	S≤8
A-14-38	S≤8	S≤4	S≤2	S≤1	S≤4	S≤4	S≤1	S≤1	S≤8	S≤8	S≤8
A-14-39	S≤8	S≤4	S≤2	S≤1	S≤4	S≤4	S≤1	S≤1	S≤8	S≤8	S≤8
A-14-40	S≤8	S≤4	S≤2	S≤1	S≤4	S≤4	S≤1	S≤1	S≤8	S≤8	S≤8
A-14-41	S≤8	S≤4	S≤2	S≤1	S≤4	S≤4	S≤1	S≤1	S≤8	S≤8	S≤8
A-14-42	R≥32	S≤4	R≥8	R≥4	S≤4	R≥16	S≤1	R≥4	S≤8	S≤8	I=16
A-14-43	R≥32	S≤4	S≤2	S≤1	S≤4	S≤4	S≤1	S≤1	S≤8	S≤8	S≤8
A-14-44	S≤8	S≤4	S≤2	S≤1	S≤4	S≤4	S≤1	S≤1	S≤8	S≤8	S≤8
A-14-45	S≤8	S≤4	S≤2	S≤1	S≤4	S≤4	S≤1	S≤1	S≤8	S≤8	S≤8
A-14-46	S≤8	S≤4	S≤2	S≤1	S≤4	S≤4	S≤1	S≤1	S≤8	S≤8	S≤8
A-14-47	S≤8	S≤4	S≤2	S≤1	S≤4	S≤4	S≤1	S≤1	S≤8	S≤8	S≤8
A-14-48	R≥32	S≤4	R≥8	S≤1	S≤4	S≤4	S≤1	S≤1	S≤8	S≤8	S≤8
A-14-49	R≥32	S≤4	R≥8	S≤1	I=8	S≤4	S≤1	S≤1	S≤8	S≤8	S≤8
A-14-50	R≥32	S≤4	R≥8	R≥4	S≤4	S≤4	S≤1	S≤1	S≤8	S≤8	S≤8
A-14-51	R≥32	S≤4	R≥8	S≤1	S≤4	S≤4	S≤1	S≤1	S≤8	S≤8	S≤8
A-14-52	S≤8	S≤4	S≤2	S≤1	S≤4	S≤4	S≤1	S≤1	S≤8	S≤8	S≤8
A-14-53	S≤8	S≤4	S≤2	S≤1	S≤4	S≤4	S≤1	S≤1	S≤8	S≤8	S≤8
A-14-54	R≥32	S≤4	R≥8	R≥4	S≤4	R≥16	S≤1	R≥4	S≤8	S≤8	I=16
A-14-55	R≥32	S≤4	R≥8	S≤1	S≤4	S≤4	S≤1	S≤1	S≤8	S≤8	S≤8
A-14-56	R≥32	S≤4	S≤2	S≤1	S≤4	S≤4	S≤1	S≤1	S≤8	S≤8	S≤8
A-14-57	R≥32	R≥16	I=4	S≤1	S≤4	S≤4	S≤1	R≥4	S≤8	S≤8	S≤8
A-14-58	R≥32	S≤4	R≥8	S≤1	S≤4	R≥16	S≤1	R≥4	S≤8	S≤8	S≤8
A-14-59	R≥32	S≤4	R≥8	R≥4	S≤4	R≥16	S≤1	R≥4	S≤8	S≤8	S≤8
A-14-60	R≥32	S≤4	I=4	S≤1	S≤4	R≥16	S≤1	R≥4	S≤8	S≤8	S≤8
A-14-61	R≥32	R≥16	S≤2	S≤1	S≤4	S≤4	S≤1	R≥4	S≤8	S≤8	S≤8
A-14-62	R≥32	S≤4	R≥8	R≥4	S≤4	R≥16	S≤1	R≥4	S≤8	S≤8	S≤8
A-14-63	S≤8	S≤4	S≤2	S≤1	S≤4	S≤4	S≤1	S≤1	S≤8	S≤8	S≤8
A-14-65	S≤8	S≤4	S≤2	S≤1	S≤4	S≤4	S≤1	S≤1	S≤8	S≤8	S≤8
A-14-66	S≤8	S≤4	S≤2	S≤1	S≤4	S≤4	S≤1	S≤1	S≤8	S≤8	S≤8
A-14-67	S≤8	S≤4	S≤2	S≤1	S≤4	S≤4	S≤1	S≤1	S≤8	S≤8	S≤8
A-14-68	S≤8	S≤4	S≤2	S≤1	S≤4	S≤4	S≤1	S≤1	S≤8	S≤8	S≤8
A-14-69	S≤8	S≤4	S≤2	S≤1	S≤4	S≤4	S≤1	S≤1	S≤8	S≤8	S≤8
A-14-70	S≤8	S≤4	S≤2	S≤1	S≤4	S≤4	S≤1	S≤1	S≤8	S≤8	S≤8
A-14-71	R≥32	S≤4	R≥8	R≥4	I=8	S≤4	S≤1	R≥4	S≤8	I=16	I=16
A-14-72	S≤8	S≤4	S≤2	S≤1	S≤4	S≤4	S≤1	S≤1	S≤8	S≤8	S≤8
A-14-73	R≥32	S≤4	R≥8	R≥4	S≤4	R≥16	S≤1	R≥4	S≤8	S≤8	S≤8
A-14-74	S≤8	S≤4	I=4	S≤1	S≤4	S≤4	S≤1	S≤1	S≤8	S≤8	S≤8
A-14-75	S≤8	S≤4	S≤2	S≤1	S≤4	S≤4	S≤1	S≤1	S≤8	S≤8	S≤8
A-14-76	S≤8	S≤4	S≤2	S≤1	S≤4	S≤4	S≤1	S≤1	S≤8	S≤8	S≤8
A-14-77	S≤8	S≤4	S≤2	S≤1	S≤4	S≤4	S≤1	S≤1	S≤8	S≤8	S≤8
A-14-78	S≤8	S≤4	S≤2	S≤1	S≤4	S≤4	S≤1	S≤1	S≤8	S≤8	S≤8
A-14-79	S≤8	S≤4	S≤2	S≤1	S≤4	S≤4	S≤1	S≤1	S≤8	S≤8	S≤8
A-14-80	R≥32	S≤4	R≥8	R≥4	S≤4	S≤4	S≤1	R≥4	S≤8	S≤8	S≤8
A-14-81	S≤8	S≤4	S≤2	S≤1	S≤4	S≤4	S≤1	S≤1	S≤8	S≤8	S≤8
A-14-82	R≥32	S≤4	R≥8	R≥4	S≤4	R≥16	S≤1	R≥4	S≤8	S≤8	S≤8
A-14-83	R≥32	S≤4	R≥8	R≥4	S≤4	R≥16	S≤1	R≥4	S≤8	R≥32	R≥32
A-14-84	R≥32	S≤4	R≥8	R≥4	S≤4	R≥16	S≤1	R≥4	S≤8	R≥32	R≥32
A-14-85	R≥32	S≤4	R≥8	R≥4	S≤4	R≥16	S≤1	R≥4	S≤8	S≤8	S≤8
A-14-86	R≥32	S≤4	R≥8	R≥4	S≤4	R≥16	S≤1	R≥4	S≤8	S≤8	S≤8
A-14-87	R≥32	S≤4	R≥8	R≥4	S≤4	R≥16	S≤1	R≥4	S≤8	S≤8	S≤8
A-14-88	R≥32	S≤4	R≥8	R≥4	S≤4	S≤4	S≤1	R≥4	S≤8	R≥32	R≥32
A-14-89	S≤8	S≤4	S≤2	S≤1	S≤4	S≤4	S≤1	S≤1	S≤8	S≤8	S≤8
A-14-90	S≤8	S≤4	S≤2	S≤1	S≤4	S≤4	S≤1	S≤1	S≤8	S≤8	S≤8
A-14-91	S≤8	S≤4	S≤2	S≤1	S≤4	S≤4	S≤1	S≤1	S≤8	S≤8	S≤8
A-14-92	R≥32	S≤4	R≥8	R≥4	S≤4	S≤4	S≤1	R≥4	S≤8	S≤8	I=16
A-14-93	S≤8	S≤4	S≤2	S≤1	S≤4	S≤4	S≤1	S≤1	S≤8	S≤8	S≤8
A-14-94	R≥32	S≤4	R≥8	R≥4	S≤4	R≥16	S≤1	R≥4	S≤8	S≤8	I=16
A-14-95	S≤8	S≤4	S≤2	S≤1	S≤4	S≤4	S≤1	S≤1	S≤8	S≤8	S≤8
A-14-96	S≤8	S≤4	S≤2	S≤1	S≤4	S≤4	S≤1	S≤1	S≤8	S≤8	S≤8
A-14-97	S≤8	S≤4	S≤2	S≤1	S≤4	S≤4	S≤1	S≤1	S≤8	S≤8	S≤8
A-14-98	S≤8	S≤4	S≤2	S≤1	S≤4	S≤4	S≤1	S≤1	S≤8	S≤8	S≤8
A-14-99	S≤8	S≤4	S≤2	S≤1	S≤4	S≤4	S≤1	S≤1	S≤8	S≤8	S≤8
A-14-100	R≥32	S≤4	R≥8	R≥4	S≤4	S≤4	S≤1	R≥4	I=16	S≤8	S≤8

→Continued on next page

→Continued from previous page

Strain code	AMP	GEN	CFZ	CTX	CAZ	TET	IMP	CIP	CHL	CPM	AZT
B-0-1	R≥32	S≤4	R≥8	R≥4	S≤4	R≥16	S≤1	S≤1	S≤8	S≤8	I=16
B-0-2	R≥32	S≤4	R≥8	R≥4	S≤4	R≥16	S≤1	S≤1	S≤8	S≤8	S≤8
B-0-3	R≥32	S≤4	R≥8	I=2	S≤4	I=8	S≤1	S≤1	I=16	S≤8	S≤8
B-0-4	R≥32	S≤4	R≥8	R≥4	S≤4	R≥16	S≤1	S≤1	S≤8	S≤8	S≤8
B-0-5	R≥32	S≤4	R≥8	S≤1	S≤4	S≤4	S≤1	S≤1	I=16	S≤8	S≤8
B-0-6	S≤8	S≤4	S≤2	S≤1	S≤4	S≤4	S≤1	S≤1	S≤8	S≤8	S≤8
B-0-7	S≤8	S≤4	S≤2	S≤1	S≤4	S≤4	S≤1	S≤1	S≤8	S≤8	S≤8
B-0-8	R≥32	S≤4	I=4	S≤1	S≤4	S≤4	S≤1	S≤1	S≤8	S≤8	S≤8
B-0-9	R≥32	S≤4	I=4	S≤1	S≤4	S≤4	S≤1	S≤1	S≤8	S≤8	S≤8
B-0-10	R≥32	S≤4	R≥8	S≤1	S≤4	S≤4	S≤1	R≥4	S≤8	S≤8	S≤8
B-0-11	S≤8	S≤4	S≤2	S≤1	S≤4	S≤4	S≤1	S≤1	S≤8	S≤8	S≤8
B-0-12	R≥32	S≤4	R≥8	R≥4	S≤4	S≤4	S≤1	R≥4	I=16	R≥32	I=16
B-0-13	R≥32	S≤4	R≥8	R≥4	S≤4	S≤4	S≤1	R≥4	S≤8	S≤8	S≤8
B-0-14	R≥32	S≤4	R≥8	R≥4	S≤4	R≥16	S≤1	R≥4	S≤8	S≤8	S≤8
B-0-15	R≥32	S≤4	S≤2	S≤1	S≤4	S≤4	S≤1	S≤1	S≤8	S≤8	S≤8
B-0-16	R≥32	S≤4	I=4	S≤1	S≤4	R≥16	S≤1	R≥4	S≤8	S≤8	S≤8
B-0-17	R≥32	S≤4	I=4	S≤1	S≤4	R≥16	S≤1	R≥4	S≤8	S≤8	S≤8
B-0-18	R≥32	S≤4	R≥8	S≤1	S≤4	R≥16	S≤1	R≥4	S≤8	S≤8	S≤8
B-0-19	R≥32	S≤4	R≥8	S≤1	S≤4	R≥16	S≤1	R≥4	S≤8	S≤8	S≤8
B-0-20	I=16	S≤4	R≥8	S≤1	S≤4	S≤4	S≤1	S≤1	S≤8	S≤8	S≤8
B-0-21	R≥32	R≥16	R≥8	S≤1	S≤4	R≥16	S≤1	R≥4	R≥32	S≤8	S≤8
B-0-22	R≥32	R≥16	R≥8	S≤1	S≤4	R≥16	S≤1	R≥4	R≥32	S≤8	S≤8
B-0-23	R≥32	S≤4	R≥8	R≥4	S≤4	R≥16	S≤1	S≤1	S≤8	I=16	I=16
B-0-24	R≥32	S≤4	R≥8	R≥4	S≤4	R≥16	S≤1	S≤1	S≤8	S≤8	S≤8
B-0-25	R≥32	S≤4	R≥8	R≥4	S≤4	R≥16	S≤1	S≤1	S≤8	S≤8	S≤8
B-0-26	R≥32	S≤4	R≥8	R≥4	S≤4	R≥16	S≤1	S≤1	S≤8	S≤8	S≤8
B-0-27	R≥32	R≥16	I=4	S≤1	S≤4	R≥16	S≤1	R≥4	R≥32	S≤8	S≤8
B-0-28	R≥32	S≤4	R≥8	S≤1	S≤4	S≤4	S≤1	S≤1	S≤8	S≤8	S≤8
B-0-29	R≥32	S≤4	R≥8	S≤1	S≤4	R≥16	S≤1	S≤1	S≤8	S≤8	S≤8
B-0-30	R≥32	S≤4	R≥8	S≤1	S≤4	R≥16	S≤1	R≥4	S≤8	S≤8	S≤8
B-0-31	R≥32	S≤4	R≥8	S≤1	S≤4	R≥16	S≤1	S≤1	S≤8	S≤8	S≤8
B-0-32	R≥32	S≤4	R≥8	S≤1	S≤4	R≥16	S≤1	R≥4	R≥32	S≤8	S≤8
B-0-33	R≥32	S≤4	R≥8	S≤1	S≤4	R≥16	S≤1	S≤1	R≥32	S≤8	S≤8
B-0-34	R≥32	S≤4	I=4	S≤1	S≤4	R≥16	S≤1	S≤1	R≥32	S≤8	S≤8
B-0-35	S≤8	S≤4	S≤2	S≤1	S≤4	S≤4	S≤1	S≤1	S≤8	S≤8	S≤8
B-0-36	S≤8	S≤4	S≤2	S≤1	S≤4	S≤4	S≤1	S≤1	S≤8	S≤8	S≤8
B-0-37	S≤8	S≤4	S≤2	S≤1	S≤4	S≤4	S≤1	S≤1	S≤8	S≤8	S≤8
B-0-38	R≥32	R≥16	R≥8	R≥4	S≤4	S≤4	S≤1	R≥4	S≤8	R≥32	I=16
B-0-39	R≥32	R≥16	R≥8	R≥4	S≤4	S≤4	S≤1	R≥4	S≤8	S≤8	S≤8
B-0-40	R≥32	S≤4	I=4	S≤1	S≤4	R≥16	S≤1	S≤1	S≤8	S≤8	S≤8
B-0-41	R≥32	S≤4	S≤2	S≤1	S≤4	R≥16	S≤1	S≤1	S≤8	S≤8	S≤8
B-0-42	S≤8	S≤4	S≤2	S≤1	S≤4	S≤4	S≤1	S≤1	S≤8	S≤8	S≤8
B-0-43	R≥32	R≥16	R≥8	R≥4	S≤4	R≥16	S≤1	R≥4	S≤8	S≤8	S≤8
B-0-44	R≥32	S≤4	I=4	S≤1	S≤4	R≥16	S≤1	S≤1	R≥32	S≤8	S≤8
B-0-45	S≤8	S≤4	S≤2	S≤1	S≤4	R≥16	S≤1	S≤1	S≤8	S≤8	S≤8
B-0-46	S≤8	S≤4	S≤2	S≤1	S≤4	R≥16	S≤1	S≤1	S≤8	S≤8	S≤8
B-0-47	S≤8	S≤4	S≤2	S≤1	S≤4	R≥16	S≤1	S≤1	S≤8	S≤8	S≤8
B-0-48	S≤8	S≤4	S≤2	S≤1	S≤4	R≥16	S≤1	S≤1	S≤8	S≤8	S≤8
B-0-49	R≥32	S≤4	R≥8	S≤1	S≤4	S≤4	S≤1	S≤1	S≤8	S≤8	S≤8
B-0-50	S≤8	S≤4	S≤2	S≤1	S≤4	S≤4	S≤1	S≤1	S≤8	S≤8	S≤8
B-0-51	S≤8	S≤4	S≤2	S≤1	S≤4	S≤4	S≤1	S≤1	S≤8	S≤8	S≤8
B-0-52	S≤8	S≤4	S≤2	S≤1	S≤4	S≤4	S≤1	S≤1	S≤8	S≤8	S≤8
B-0-53	S≤8	S≤4	S≤2	S≤1	S≤4	S≤4	S≤1	S≤1	S≤8	S≤8	S≤8
B-0-54	S≤8	S≤4	S≤2	S≤1	S≤4	S≤4	S≤1	S≤1	S≤8	S≤8	S≤8
B-0-55	S≤8	S≤4	S≤2	S≤1	S≤4	S≤4	S≤1	S≤1	S≤8	S≤8	S≤8
B-0-56	S≤8	S≤4	S≤2	S≤1	S≤4	S≤4	S≤1	S≤1	S≤8	S≤8	S≤8
B-0-57	S≤8	S≤4	S≤2	S≤1	S≤4	R≥16	S≤1	S≤1	S≤8	S≤8	S≤8
B-0-58	R≥32	S≤4	R≥8	R≥4	R≥16	S≤4	S≤1	S≤1	S≤8	S≤8	I=16
B-0-59	R≥32	S≤4	R≥8	R≥4	R≥16	S≤4	S≤1	S≤1	S≤8	S≤8	I=16
B-0-60	R≥32	S≤4	R≥8	R≥4	S≤4	S≤4	S≤1	R≥4	S≤8	S≤8	S≤8
B-0-61	S≤8	S≤4	S≤2	S≤1	S≤4	S≤4	S≤1	S≤1	S≤8	S≤8	S≤8
B-0-62	R≥32	S≤4	R≥8	R≥4	S≤4	S≤4	S≤1	R≥4	S≤8	S≤8	S≤8
B-0-63	S≤8	S≤4	S≤2	S≤1	S≤4	S≤4	S≤1	S≤1	S≤8	S≤8	S≤8
B-0-64	R≥32	S≤4	R≥8	R≥4	S≤4	S≤4	S≤1	S≤1	S≤8	S≤8	S≤8
B-0-65	R≥32	S≤4	R≥8	R≥4	S≤4	S≤4	S≤1	S≤1	S≤8	S≤8	S≤8
B-0-66	R≥32	S≤4	R≥8	R≥4	S≤4	S≤4	S≤1	R≥4	S≤8	S≤8	S≤8
B-0-67	R≥32	S≤4	R≥8	R≥4	S≤4	S≤4	S≤1	R≥4	S≤8	S≤8	S≤8
B-0-68	S≤8	S≤4	S≤2	S≤1	S≤4	S≤4	S≤1	S≤1	S≤8	S≤8	S≤8
B-0-69	S≤8	S≤4	S≤2	S≤1	S≤4	R≥16	S≤1	S≤1	S≤8	S≤8	S≤8
B-0-70	S≤8	S≤4	S≤2	S≤1	S≤4	S≤4	S≤1	S≤1	S≤8	S≤8	S≤8
B-0-71	S≤8	S≤4	S≤2	S≤1	S≤4	S≤4	S≤1	S≤1	S≤8	S≤8	S≤8
B-0-72	R≥32	S≤4	S≤2	S≤1	S≤4	R≥16	S≤1	S≤1	S≤8	S≤8	S≤8
B-0-73	R≥32	S≤4	S≤2	S≤1	S≤4	R≥16	S≤1	S≤1	S≤8	S≤8	S≤8
B-0-74	R≥32	S≤4	S≤2	S≤1	S≤4	R≥16	S≤1	S≤1	S≤8	S≤8	S≤8
B-0-75	R≥32	S≤4	S≤2	S≤1	S≤4	R≥16	S≤1	R≥4	S≤8	S≤8	S≤8
B-0-76	S≤8	S≤4	S≤2	S≤1	S≤4	I=8	S≤1	R≥4	S≤8	S≤8	S≤8
B-0-77	S≤8	S≤4	S≤2	S≤1	S≤4	S≤4	S≤1	S≤1	S≤8	S≤8	S≤8
B-0-78	S≤8	S≤4	S≤2	S≤1	S≤4	S≤4	S≤1	S≤1	S≤8	S≤8	S≤8
B-0-79	R≥32	S≤4	R≥8	R≥4	R≥16	S≤4	S≤1	S≤1	S≤8	S≤8	R≥32
B-0-80	R≥32	S≤4	R≥8	R≥4	R≥16	S≤4	S≤1	S≤1	S≤8	S≤8	I=16
B-0-81	R≥32	S≤4	R≥8	R≥4	R≥16	S≤4	S≤1	S≤1	S≤8	S≤8	I=16
B-0-82	S≤8	S≤4	S≤2	S≤1	S≤4	S≤4	S≤1	S≤1	S≤8	S≤8	S≤8
B-0-83	S≤8	S≤4	S≤2	S≤1	S≤4	S≤4	S≤1	S≤1	S≤8	S≤8	S≤8
B-0-84	S≤8	S≤4	S≤2	S≤1	S≤4	S≤4	S≤1	S≤1	S≤8	S≤8	S≤8
B-0-85	R≥32	S≤4	R≥8	R≥4	S≤4	S≤4	S≤1	R≥4	S≤8	I=16	I=16
B-0-86	R≥32	S≤4	R≥8	R≥4	S≤4	S≤4	S≤1	R≥4	S≤8	S≤8	S≤8
B-0-87	R≥32	S≤4	R≥8	R≥4	S≤4	S≤4	S≤1	R≥4	S≤8	S≤8	S≤8
B-0-88	R≥32	S≤4	R≥8	R≥4	S≤4	S≤4	S≤1	R≥4	S≤8	S≤8	S≤8
B-0-89	R≥32	S≤4	R≥8	R≥4	S≤4	S≤4	S≤1	R≥4	S≤8	S≤8	S≤8
B-0-90	R≥32	S≤4	R≥8	S≤1	S≤4	S≤4	S≤1	S≤1	S≤8	S≤8	S≤8
B-0-91	S≤8	S≤4	S≤2	S≤1	S≤4	S≤4	S≤1	S≤1	S≤8	S≤8	S≤8
B-0-92	R≥32	S≤4	R≥8	S≤1	S≤4	S≤4	S≤1	S≤1	S≤8	S≤8	S≤8
B-0-93	S≤8	S≤4	S≤2	S≤1	S≤4	S≤4	S≤1	S≤1	S≤8	S≤8	S≤8
B-0-94	S≤8	S≤4	S≤2	S≤1	S≤4	S≤4	S≤1	S≤1	S≤8	S≤8	S≤8
B-0-95	S≤8	S≤4	S≤2	S≤1	S≤4	S≤4	S≤1	S≤1	S≤8	S≤8	S≤8
B-0-96	S≤8	S≤4	S≤2	S≤1	S≤4	S≤4	S≤1	S≤1	S≤8	S≤8	S≤8
B-0-97	S≤8	S≤4	S≤2	S≤1	S≤4	S≤4	S≤1	S≤1	S≤8	S≤8	S≤8
B-0-98	S≤8	S≤4	S≤2	S≤1	S≤4	S≤4	S≤1	S≤1	S≤8	S≤8	S≤8
B-0-99	R≥32	S≤4	S≤2	S≤1	S≤4	R≥16	S≤1	S≤1	S≤8	S≤8	S≤8
B-0-100	R≥32	S≤4	S≤2	S≤1	S≤4	R≥16	S≤1	S≤1	S≤8	S≤8	S≤8

Strain code	AMP	GEN	CFZ	CTX	CAZ	TET	IMP	CIP	CHL	CPM	AZT
B-7-1	S≤8	S≤4	S≤2	S≤1	S≤4	S≤4	S≤1	S≤1	S≤8	S≤8	S≤8
B-7-2	R≥32	S≤4	R≥8	R≥4	R≥16	S≤4	S≤1	S≤1	S≤8	S≤8	S≤8
B-7-3	S≤8	S≤4	S≤2	S≤1	S≤4	S≤4	S≤1	S≤1	S≤8	S≤8	S≤8
B-7-4	I=16	S≤4	S≤2	I=2	S≤4	S≤4	S≤1	S≤1	S≤8	S≤8	S≤8
B-7-5	S≤8	S≤4	S≤2	S≤1	S≤4	S≤4	S≤1	S≤1	S≤8	S≤8	S≤8
B-7-6	S≤8	S≤4	S≤2	S≤1	S≤4	S≤4	S≤1	S≤1	S≤8	S≤8	S≤8
B-7-7	S≤8	S≤4	S≤2	S≤1	S≤4	S≤4	S≤1	S≤1	S≤8	S≤8	S≤8
B-7-8	R≥32	S≤4	S≤2	S≤1	S≤4	R≥16	S≤1	S≤1	S≤8	S≤8	S≤8
B-7-9	S≤8	S≤4	S≤2	S≤1	S≤4	S≤4	S≤1	R≥4	S≤8	S≤8	S≤8
B-7-10	R≥32	S≤4	S≤2	S≤1	S≤4	R≥16	S≤1	S≤1	S≤8	S≤8	S≤8
B-7-11	S≤8	S≤4	S≤2	S≤1	S≤4	R≥16	S≤1	S≤1	S≤8	S≤8	S≤8
B-7-12	R≥32	S≤4	S≤2	S≤1	S≤4	R≥16	S≤1	R≥4	S≤8	S≤8	S≤8
B-7-13	S≤8	S≤4	S≤2	S≤1	S≤4	R≥16	S≤1	S≤1	S≤8	S≤8	S≤8
B-7-14	S≤8	S≤4	S≤2	S≤1	S≤4	S≤4	S≤1	S≤1	S≤8	S≤8	S≤8
B-7-15	R≥32	S≤4	S≤2	S≤1	S≤4	R≥16	S≤1	S≤1	S≤8	S≤8	S≤8
B-7-16	R≥32	S≤4	S≤2	S≤1	S≤4	R≥16	S≤1	S≤1	S≤8	S≤8	S≤8
B-7-17	R≥32	S≤4	R≥8	R≥4	S≤4	R≥16	S≤1	S≤1	S≤8	S≤8	S≤8
B-7-18	R≥32	S≤4	R≥8	R≥4	S≤4	R≥16	S≤1	S≤1	S≤8	S≤8	S≤8
B-7-19	R≥32	S≤4	R≥8	R≥4	S≤4	R≥16	S≤1	S≤1	S≤8	S≤8	S≤8
B-7-20	R≥32	S≤4	R≥8	R≥4	S≤4	R≥16	S≤1	S≤1	S≤8	S≤8	S≤8
B-7-21	R≥32	S≤4	R≥8	S≤1	S≤4	R≥16	S≤1	R≥4	S≤8	S≤8	S≤8
B-7-22	S≤8	S≤4	R≥8	S≤1	S≤4	S≤4	S≤1	S≤1	S≤8	S≤8	S≤8
B-7-23	S≤8	S≤4	S≤2	S≤1	S≤4	R≥16	S≤1	S≤1	S≤8	S≤8	S≤8
B-7-24	R≥32	S≤4	S≤2	S≤1	S≤4	R≥16	S≤1	S≤1	S≤8	S≤8	S≤8
B-7-25	S≤8	S≤4	S≤2	S≤1	S≤4	S≤4	S≤1	S≤1	S≤8	S≤8	S≤8
B-7-26	R≥32	S≤4	R≥8	R≥4	S≤4	R≥16	S≤1	S≤1	S≤8	S≤8	S≤8
B-7-27	S≤8	S≤4	S≤2	S≤1	S≤4	S≤4	S≤1	S≤1	R≥32	S≤8	S≤8
B-7-28	S≤8	S≤4	S≤2	S≤1	S≤4	S≤4	S≤1	S≤1	S≤8	S≤8	S≤8
B-7-29	S≤8	S≤4	S≤2	S≤1	S≤4	S≤4	S≤1	S≤1	S≤8	S≤8	S≤8
B-7-30	S≤8	S≤4	R≥8	S≤1	S≤4	S≤4	S≤1	S≤1	S≤8	S≤8	S≤8
B-7-31	R≥32	S≤4	S≤2	S≤1	S≤4	R≥16	S≤1	S≤1	S≤8	S≤8	S≤8
B-7-32	R≥32	S≤4	R≥8	R≥4	R≥16	S≤4	S≤1	S≤1	S≤8	S≤8	S≤8
B-7-33	R≥32	S≤4	R≥8	R≥4	I=8	R≥16	S≤1	S≤1	S≤8	S≤8	S≤8
B-7-34	R≥32	S≤4	R≥8	R≥4	I=8	R≥16	S≤1	S≤1	S≤8	S≤8	S≤8
B-7-35	R≥32	S≤4	R≥8	R≥4	I=8	R≥16	S≤1	S≤1	S≤8	S≤8	S≤8
B-7-36	R≥32	S≤4	S≤2	S≤1	S≤4	R≥16	S≤1	S≤1	S≤8	S≤8	S≤8
B-7-37	R≥32	S≤4	S≤2	S≤1	S≤4	R≥16	S≤1	S≤1	S≤8	S≤8	S≤8
B-7-38	R≥32	S≤4	S≤2	I=2	S≤4	R≥16	S≤1	S≤1	S≤8	S≤8	S≤8
B-7-39	R≥32	S≤4	S≤2	S≤1	S≤4	R≥16	S≤1	S≤1	S≤8	S≤8	S≤8
B-7-40	R≥32	S≤4	R≥8	R≥4	S≤4	R≥16	S≤1	R≥4	S≤8	S≤8	R
B-7-41	S≤8	S≤4	S≤2	S≤1	S≤4	S≤4	S≤1	S≤1	S≤8	S≤8	S≤8
B-7-42	I=16	S≤4	R≥8	S≤1	S≤4	S≤4	S≤1	S≤1	S≤8	S≤8	S≤8
B-7-43	R≥32	S≤4	R≥8	S≤1	S≤4	R≥16	S≤1	S≤1	S≤8	S≤8	S≤8
B-7-44	I=16	S≤4	R≥8	S≤1	S≤4	S≤4	S≤1	S≤1	S≤8	S≤8	S≤8
B-7-45	R≥32	S≤4	R≥8	R≥4	S≤4	R≥16	S≤1	S≤1	S≤8	S≤8	S≤8
B-7-46	R≥32	S≤4	R≥8	R≥4	S≤4	R≥16	S≤1	S≤1	S≤8	S≤8	I=16
B-7-47	R≥32	S≤4	R≥8	R≥4	S≤4	R≥16	S≤1	S≤1	S≤8	S≤8	S≤8
B-7-48	R≥32	S≤4	R≥8	R≥4	S≤4	R≥16	S≤1	S≤1	S≤8	S≤8	S≤8
B-7-49	R≥32	S≤4	R≥8	R≥4	S≤4	R≥16	S≤1	R≥4	S≤8	S≤8	I=16
B-7-50	R≥32	S≤4	R≥8	R≥4	S≤4	R≥16	S≤1	R≥4	S≤8	S≤8	I=16
B-7-51	R≥32	S≤4	R≥8	R≥4	I=8	R≥16	S≤1	S≤1	S≤8	S≤8	R≥32
B-7-52	R≥32	S≤4	S≤2	S≤1	S≤4	R≥16	S≤1	S≤1	S≤8	S≤8	S≤8
B-7-53	R≥32	S≤4	R≥8	R≥4	S≤4	R≥16	S≤1	S≤1	S≤8	S≤8	S≤8
B-7-54	R≥32	S≤4	S≤2	S≤1	S≤4	R≥16	S≤1	S≤1	S≤8	S≤8	S≤8
B-7-55	R≥32	S≤4	S≤2	S≤1	S≤4	R≥16	S≤1	S≤1	S≤8	S≤8	S≤8
B-7-56	R≥32	S≤4	R≥8	S≤1	S≤4	R≥16	S≤1	S≤1	R≥32	S≤8	S≤8
B-7-57	S≤8	S≤4	S≤2	S≤1	S≤4	S≤4	S≤1	S≤1	S≤8	S≤8	S≤8
B-7-58	R≥32	S≤4	R≥8	R≥4	S≤4	R≥16	S≤1	S≤1	S≤8	S≤8	S≤8
B-7-59	S≤8	S≤4	R≥8	S≤1	S≤4	S≤4	S≤1	S≤1	S≤8	S≤8	S≤8
B-7-60	S≤8	S≤4	R≥8	S≤1	S≤4	S≤4	S≤1	S≤1	S≤8	S≤8	S≤8
B-7-61	S≤8	S≤4	R≥8	S≤1	S≤4	R≥16	S≤1	S≤1	S≤8	S≤8	S≤8
B-7-62	S≤8	S≤4	S≤2	S≤1	S≤4	R≥16	S≤1	R≥4	R≥32	S≤8	S≤8
B-7-63	R≥32	S≤4	I=4	S≤1	S≤4	R≥16	S≤1	R≥4	R≥32	S≤8	S≤8
B-7-64	R≥32	S≤4	S≤2	S≤1	S≤4	R≥16	S≤1	R≥4	R≥32	S≤8	S≤8
B-7-65	I=16	S≤4	R≥8	S≤1	S≤4	S≤4	S≤1	S≤1	S≤8	S≤8	S≤8
B-7-66	S≤8	R≥16	R≥8	S≤1	S≤4	R≥16	S≤1	R≥4	S≤8	S≤8	S≤8
B-7-67	R≥32	S≤4	R≥8	R≥4	S≤4	R≥16	S≤1	S≤1	S≤8	S≤8	S≤8
B-7-68	R≥32	S≤4	R≥8	R≥4	S≤4	R≥16	S≤1	S≤1	S≤8	S≤8	I=16
B-7-69	R≥32	S≤4	R≥8	R≥4	S≤4	R≥16	S≤1	S≤1	S≤8	S≤8	S≤8
B-7-70	R≥32	S≤4	R≥8	R≥4	S≤4	R≥16	S≤1	S≤1	S≤8	S≤8	S≤8
B-7-71	R≥32	S≤4	R≥8	R≥4	S≤4	R≥16	S≤1	S≤1	S≤8	S≤8	S≤8
B-7-72	I=16	S≤4	R≥8	S≤1	S≤4	S≤4	S≤1	S≤1	S≤8	S≤8	S≤8
B-7-73	S≤8	S≤4	S≤2	S≤1	S≤4	S≤4	S≤1	S≤1	S≤8	S≤8	S≤8
B-7-74	S≤8	S≤4	S≤2	S≤1	S≤4	S≤4	S≤1	S≤1	S≤8	S≤8	S≤8
B-7-75	S≤8	S≤4	S≤2	S≤1	S≤4	S≤4	S≤1	S≤1	S≤8	S≤8	S≤8
B-7-76	S≤8	S≤4	S≤2	S≤1	S≤4	S≤4	S≤1	S≤1	S≤8	S≤8	S≤8
B-7-77	R≥32	S≤4	S≤2	S≤1	S≤4	R≥16	S≤1	S≤1	S≤8	S≤8	S≤8
B-7-78	R≥32	S≤4	S≤2	S≤1	S≤4	R≥16	S≤1	S≤1	S≤8	S≤8	S≤8
B-7-79	R≥32	S≤4	S≤2	S≤1	S≤4	R≥16	S≤1	S≤1	S≤8	S≤8	S≤8
B-7-80	R≥32	S≤4	R≥8	S≤1	S≤4	R≥16	S≤1	R≥4	S≤8	S≤8	S≤8
B-7-81	R≥32	S≤4	S≤2	S≤1	S≤4	R≥16	S≤1	S≤1	S≤8	S≤8	S≤8
B-7-82	S≤8	S≤4	S≤2	S≤1	S≤4	S≤4	S≤1	S≤1	S≤8	S≤8	S≤8
B-7-83	S≤8	S≤4	R≥8	S≤1	S≤4	S≤4	S≤1	S≤1	S≤8	S≤8	S≤8
B-7-84	R≥32	R≥16	R≥8	R≥4	S≤4	S≤4	S≤1	R≥4	S≤8	S≤8	S≤8
B-7-85	S≤8	S≤4	S≤2	S≤1	S≤4	R≥16	S≤1	S≤1	S≤8	S≤8	S≤8
B-7-86	S≤8	S≤4	R≥8	S≤1	S≤4	S≤4	S≤1	S≤1	S≤8	S≤8	S≤8
B-7-87	R≥32	S≤4	R≥8	S≤1	S≤4	R≥16	S≤1	S≤1	R≥32	S≤8	S≤8
B-7-88	R≥32	R≥16	R≥8	R≥4	S≤4	S≤4	S≤1	R≥4	S≤8	S≤8	S≤8
B-7-89	R≥32	S≤4	R≥8	S≤1	S≤4	S≤4	S≤1	S≤1	S≤8	S≤8	S≤8
B-7-90	R≥32	S≤4	S≤2	S≤1	S≤4	S≤4	S≤1	S≤1	S≤8	S≤8	S≤8
B-7-91	R≥32	S≤4	S≤2	S≤1	S≤4	R≥16	S≤1	S≤1	R≥32	S≤8	S≤8
B-7-92	R≥32	S≤4	S≤2	S≤1	S≤4	S≤4	S≤1	S≤1	S≤8	S≤8	S≤8
B-7-93	R≥32	S≤4	R≥8	S≤1	S≤4	R≥16	S≤1	S≤1	I=16	S≤8	S≤8
B-7-94	S≤8	S≤4	S≤2	S≤1	S≤4	S≤4	S≤1	S≤1	S≤8	S≤8	S≤8
B-7-95	S≤8	S≤4	I=4	S≤1	S≤4	S≤4	S≤1	S≤1	S≤8	S≤8	S≤8
B-7-96	S≤8	S≤4	S≤2	S≤1	S≤4	S≤4	S≤1	S≤1	S≤8	S≤8	S≤8
B-7-97	S≤8	S≤4	S≤2	S≤1	S≤4	R≥16	S≤1	S≤1	S≤8	S≤8	S≤8
B-7-98	S≤8	S≤4	S≤2	S≤1	S≤4	R≥16	S≤1	S≤1	S≤8	S≤8	S≤8
B-7-99	S≤8	S≤4	S≤2	R≥4	S≤4	R≥16	S≤1	S≤1	S≤8	S≤8	S≤8
B-7-100	S≤8	S≤4	S≤2	S≤1	S≤4	S≤4	S≤1	S≤1	S≤8	S≤8	S≤8

Strain code	AMP	GEN	CFZ	CTX	CAZ	TET	IMP	CIP	CHL	CPM	AZT
B-14-1	S≤8	S≤4	S≤2	S≤1	S≤4	S≤4	S≤1	S≤1	S≤8	S≤8	S≤8
B-14-2	S≤8	S≤4	S≤2	S≤1	S≤4	S≤4	S≤1	S≤1	S≤8	S≤8	S≤8
B-14-3	R≥32	S≤4	S≤2	S≤1	S≤4	R≥16	S≤1	R≥4	S≤8	S≤8	S≤8
B-14-4	S≤8	S≤4	S≤2	S≤1	S≤4	S≤4	S≤1	S≤1	S≤8	S≤8	S≤8
B-14-5	R≥32	S≤4	S≤2	S≤1	S≤4	R≥16	S≤1	R≥4	S≤8	S≤8	S≤8
B-14-6	S≤8	S≤4	S≤2	S≤1	S≤4	S≤4	S≤1	S≤1	S≤8	S≤8	S≤8
B-14-7	S≤8	S≤4	S≤2	S≤1	S≤4	S≤4	S≤1	S≤1	S≤8	S≤8	S≤8
B-14-8	R≥32	S≤4	S≤2	S≤1	S≤4	S≤4	S≤1	S≤1	S≤8	S≤8	S≤8
B-14-9	S≤8	S≤4	S≤2	S≤1	S≤4	S≤4	S≤1	S≤1	S≤8	S≤8	S≤8
B-14-10	S≤8	S≤4	S≤2	S≤1	S≤4	S≤4	S≤1	S≤1	S≤8	S≤8	S≤8
B-14-11	S≤8	S≤4	S≤2	S≤1	S≤4	S≤4	S≤1	S≤1	S≤8	S≤8	S≤8
B-14-12	S≤8	S≤4	S≤2	S≤1	S≤4	S≤4	S≤1	S≤1	S≤8	S≤8	S≤8
B-14-13	S≤8	S≤4	S≤2	S≤1	S≤4	S≤4	S≤1	S≤1	S≤8	S≤8	S≤8
B-14-14	S≤8	S≤4	S≤2	S≤1	S≤4	R≥16	S≤1	S≤1	S≤8	S≤8	S≤8
B-14-15	S≤8	S≤4	S≤2	S≤1	S≤4	R≥16	S≤1	S≤1	S≤8	S≤8	S≤8
B-14-16	R≥32	S≤4	R≥8	S≤1	S≤4	R≥16	S≤1	S≤1	S≤8	S≤8	S≤8
B-14-17	S≤8	S≤4	S≤2	S≤1	S≤4	S≤4	S≤1	S≤1	S≤8	S≤8	S≤8
B-14-18	S≤8	S≤4	S≤2	S≤1	S≤4	S≤4	S≤1	S≤1	S≤8	S≤8	S≤8
B-14-19	S≤8	S≤4	S≤2	S≤1	S≤4	R≥16	S≤1	S≤1	S≤8	S≤8	S≤8
B-14-20	S≤8	S≤4	S≤2	S≤1	S≤4	R≥16	S≤1	S≤1	S≤8	S≤8	S≤8
B-14-21	S≤8	S≤4	S≤2	S≤1	S≤4	R≥16	S≤1	S≤1	S≤8	S≤8	S≤8
B-14-22	S≤8	S≤4	S≤2	S≤1	S≤4	S≤4	S≤1	S≤1	S≤8	S≤8	S≤8
B-14-23	S≤8	S≤4	S≤2	S≤1	S≤4	S≤4	S≤1	S≤1	S≤8	S≤8	S≤8
B-14-24	S≤8	S≤4	S≤2	S≤1	S≤4	S≤4	S≤1	S≤1	S≤8	S≤8	S≤8
B-14-25	S≤8	S≤4	S≤2	S≤1	S≤4	S≤4	S≤1	S≤1	S≤8	S≤8	S≤8
B-14-26	S≤8	S≤4	S≤2	S≤1	S≤4	S≤4	S≤1	S≤1	S≤8	S≤8	S≤8
B-14-27	R≥32	S≤4	R≥8	R≥4	S≤4	R≥16	S≤1	R≥4	S≤8	S≤8	S≤8
B-14-28	R≥32	S≤4	R≥8	R≥4	S≤4	R≥16	S≤1	R≥4	S≤8	S≤8	S≤8
B-14-29	S≤8	S≤4	S≤2	S≤1	S≤4	S≤4	S≤1	S≤1	S≤8	S≤8	S≤8
B-14-30	R≥32	S≤4	R≥8	R≥4	S≤4	R≥16	S≤1	R≥4	S≤8	S≤8	S≤8
B-14-31	R≥32	S≤4	R≥8	S≤1	S≤4	S≤4	S≤1	S≤1	S≤8	S≤8	S≤8
B-14-32	S≤8	S≤4	S≤2	S≤1	S≤4	S≤4	S≤1	S≤1	S≤8	S≤8	S≤8
B-14-33	R≥32	S≤4	R≥8	R≥4	S≤4	R≥16	S≤1	R≥4	S≤8	S≤8	S≤8
B-14-34	R≥32	S≤4	R≥8	R≥4	S≤4	R≥16	S≤1	R≥4	S≤8	S≤8	S≤8
B-14-35	S≤8	S≤4	S≤2	S≤1	S≤4	R≥16	S≤1	S≤1	S≤8	S≤8	S≤8
B-14-36	R≥32	S≤4	R≥8	R≥4	S≤4	R≥16	S≤1	R≥4	S≤8	S≤8	S≤8
B-14-37	R≥32	S≤4	R≥8	R≥4	S≤4	R≥16	S≤1	R≥4	S≤8	S≤8	S≤8
B-14-38	S≤8	S≤4	S≤2	S≤1	S≤4	R≥16	S≤1	S≤1	S≤8	S≤8	S≤8
B-14-39	R≥32	S≤4	R≥8	R≥4	S≤4	R≥16	S≤1	R≥4	S≤8	S≤8	S≤8
B-14-40	S≤8	S≤4	S≤2	S≤1	S≤4	R≥16	S≤1	S≤1	S≤8	S≤8	S≤8
B-14-41	S≤8	S≤4	S≤2	S≤1	S≤4	R≥16	S≤1	S≤1	S≤8	S≤8	S≤8
B-14-42	R≥32	S≤4	R≥8	R≥4	S≤4	R≥16	S≤1	R≥4	S≤8	S≤8	S≤8
B-14-43	S≤8	S≤4	S≤2	S≤1	S≤4	S≤4	S≤1	S≤1	S≤8	S≤8	S≤8
B-14-44	R≥32	R≥16	S≤2	S≤1	S≤4	R≥16	S≤1	S≤1	S≤8	S≤8	S≤8
B-14-45	R≥32	R≥16	R≥8	S≤1	S≤4	R≥16	S≤1	R≥4	S≤8	S≤8	S≤8
B-14-46	R≥32	R≥16	S≤2	S≤1	S≤4	R≥16	S≤1	R≥4	S≤8	S≤8	S≤8
B-14-47	R≥32	R≥16	S≤2	S≤1	S≤4	R≥16	S≤1	R≥4	S≤8	S≤8	S≤8
B-14-48	S≤8	S≤4	S≤2	S≤1	S≤4	S≤4	S≤1	S≤1	S≤8	S≤8	S≤8
B-14-49	S≤8	S≤4	S≤2	S≤1	S≤4	S≤4	S≤1	S≤1	S≤8	S≤8	S≤8
B-14-50	R≥32	S≤4	R≥8	S≤1	S≤4	S≤4	S≤1	S≤1	S≤8	S≤8	S≤8
B-14-51	R≥32	S≤4	R≥8	S≤1	S≤4	S≤4	S≤1	S≤1	S≤8	S≤8	S≤8
B-14-52	R≥32	S≤4	R≥8	S≤1	S≤4	S≤4	S≤1	S≤1	S≤8	S≤8	S≤8
B-14-53	S≤8	S≤4	S≤2	S≤1	S≤4	S≤4	S≤1	S≤1	S≤8	S≤8	S≤8
B-14-54	R≥32	S≤4	R≥8	S≤1	S≤4	S≤4	S≤1	S≤1	S≤8	S≤8	S≤8
B-14-55	R≥32	S≤4	R≥8	S≤1	S≤4	S≤4	S≤1	S≤1	S≤8	S≤8	S≤8
B-14-56	S≤8	S≤4	S≤2	S≤1	S≤4	S≤4	S≤1	S≤1	S≤8	S≤8	S≤8
B-14-57	S≤8	S≤4	S≤2	S≤1	S≤4	S≤4	S≤1	S≤1	S≤8	S≤8	S≤8
B-14-58	R≥32	R≥16	S≤2	S≤1	S≤4	R≥16	S≤1	S≤1	S≤8	S≤8	S≤8
B-14-59	S≤8	S≤4	S≤2	S≤1	S≤4	S≤4	S≤1	S≤1	S≤8	S≤8	S≤8
B-14-60	S≤8	S≤4	S≤2	S≤1	S≤4	S≤4	S≤1	S≤1	S≤8	S≤8	S≤8
B-14-61	S≤8	S≤4	S≤2	S≤1	S≤4	S≤4	S≤1	S≤1	S≤8	S≤8	S≤8
B-14-62	S≤8	S≤4	S≤2	S≤1	S≤4	S≤4	S≤1	S≤1	S≤8	S≤8	S≤8
B-14-63	S≤8	S≤4	S≤2	S≤1	S≤4	S≤4	S≤1	S≤1	S≤8	S≤8	S≤8
B-14-64	S≤8	S≤4	S≤2	S≤1	S≤4	S≤4	S≤1	S≤1	S≤8	S≤8	S≤8
B-14-65	R≥32	R≥16	S≤2	S≤1	S≤4	R≥16	S≤1	S≤1	S≤8	S≤8	S≤8
B-14-66	R≥32	R≥16	S≤2	S≤1	S≤4	S≤4	S≤1	R≥4	S≤8	S≤8	S≤8
B-14-67	R≥32	R≥16	S≤2	S≤1	S≤4	R≥16	S≤1	S≤1	S≤8	S≤8	S≤8
B-14-68	R≥32	R≥16	S≤2	S≤1	S≤4	R≥16	S≤1	S≤1	S≤8	S≤8	S≤8
B-14-69	S≤8	S≤4	S≤2	S≤1	S≤4	S≤4	S≤1	S≤1	S≤8	S≤8	S≤8
B-14-70	R≥32	S≤4	I=4	S≤1	S≤4	R≥16	S≤1	S≤1	S≤8	S≤8	S≤8
B-14-71	S≤8	S≤4	S≤2	S≤1	S≤4	S≤4	S≤1	S≤1	S≤8	S≤8	S≤8
B-14-72	S≤8	S≤4	S≤2	S≤1	S≤4	S≤4	S≤1	S≤1	S≤8	S≤8	S≤8
B-14-73	R≥32	S≤4	R≥8	S≤1	S≤4	S≤4	S≤1	S≤1	S≤8	S≤8	S≤8
B-14-74	S≤8	S≤4	S≤2	S≤1	S≤4	S≤4	S≤1	S≤1	S≤8	S≤8	S≤8
B-14-75	S≤8	S≤4	S≤2	S≤1	S≤4	S≤4	S≤1	S≤1	S≤8	S≤8	S≤8
B-14-76	S≤8	S≤4	S≤2	S≤1	S≤4	S≤4	S≤1	S≤1	S≤8	S≤8	S≤8
B-14-77	S≤8	S≤4	S≤2	S≤1	S≤4	S≤4	S≤1	S≤1	S≤8	S≤8	S≤8
B-14-78	R≥32	S≤4	R≥8	S≤1	S≤4	S≤4	S≤1	R≥4	S≤8	S≤8	S≤8
B-14-79	S≤8	S≤4	S≤2	S≤1	S≤4	S≤4	S≤1	S≤1	S≤8	S≤8	S≤8
B-14-80	R≥32	S≤4	S≤2	S≤1	S≤4	R≥16	S≤1	S≤1	S≤8	S≤8	S≤8
B-14-81	S≤8	S≤4	S≤2	S≤1	S≤4	S≤4	S≤1	S≤1	S≤8	S≤8	S≤8
B-14-82	S≤8	S≤4	S≤2	S≤1	S≤4	S≤4	S≤1	S≤1	S≤8	S≤8	S≤8
B-14-83	R≥32	S≤4	S≤2	S≤1	S≤4	R≥16	S≤1	S≤1	S≤8	S≤8	S≤8
B-14-84	S≤8	S≤4	S≤2	S≤1	S≤4	S≤4	S≤1	S≤1	S≤8	S≤8	S≤8
B-14-85	S≤8	S≤4	S≤2	S≤1	S≤4	S≤4	S≤1	S≤1	S≤8	S≤8	S≤8
B-14-86	S≤8	S≤4	S≤2	S≤1	S≤4	S≤4	S≤1	S≤1	S≤8	S≤8	S≤8
B-14-87	S≤8	S≤4	S≤2	S≤1	S≤4	S≤4	S≤1	S≤1	S≤8	S≤8	S≤8
B-14-88	R≥32	S≤4	S≤2	S≤1	S≤4	R≥16	S≤1	S≤1	S≤8	S≤8	S≤8
B-14-89	S≤8	S≤4	S≤2	S≤1	S≤4	S≤4	S≤1	S≤1	S≤8	S≤8	S≤8
B-14-90	S≤8	S≤4	S≤2	S≤1	S≤4	S≤4	S≤1	R≥4	S≤8	S≤8	S≤8
B-14-91	S≤8	S≤4	S≤2	S≤1	S≤4	S≤4	S≤1	R≥4	S≤8	S≤8	S≤8
B-14-92	S≤8	S≤4	S≤2	S≤1	S≤4	S≤4	S≤1	R≥4	S≤8	S≤8	S≤8
B-14-93	S≤8	S≤4	S≤2	S≤1	S≤4	S≤4	S≤1	S≤1	S≤8	S≤8	S≤8
B-14-94	S≤8	S≤4	S≤2	S≤1	S≤4	S≤4	S≤1	S≤1	S≤8	S≤8	S≤8
B-14-95	S≤8	S≤4	S≤2	S≤1	S≤4	S≤4	S≤1	S≤1	S≤8	S≤8	S≤8
B-14-96	S≤8	S≤4	S≤2	S≤1	S≤4	R≥16	S≤1	S≤1	S≤8	S≤8	S≤8
B-14-97	S≤8	S≤4	S≤2	S≤1	S≤4	S≤4	S≤1	S≤1	S≤8	S≤8	S≤8
B-14-98	S≤8	S≤4	S≤2	S≤1	S≤4	S≤4	S≤1	S≤1	S≤8	S≤8	S≤8
B-14-99	S≤8	S≤4	S≤2	S≤1	S≤4	S≤4	S≤1	S≤1	S≤8	S≤8	S≤8
B-14-100	S≤8	S≤4	S≤2	S≤1	S≤4	S≤4	S≤1	R≥4	S≤8	S≤8	S≤8
